# Characterizing the microstructural organization of collagenous fibrils in articular cartilage extracellular matrix using diffusion tensor imaging: A cross-sectional observational study

**DOI:** 10.1097/MD.0000000000042902

**Published:** 2025-06-20

**Authors:** Jie Liu, Xiaomu Tang

**Affiliations:** a Radiology Department, Wuhan Fourth Hospital, Wuhan, China.

**Keywords:** articular cartilage, collagen, diffusion tensor imaging, extracellular matrix, knee pain, osteoarthritis

## Abstract

In the exploration of articular cartilage extracellular matrix (ECM) microstructural organization, the use of diffusion tensor imaging (DTI) has emerged as a promising noninvasive imaging technique. The study aimed to characterize the collagenous fibril architecture within the ECM of articular cartilage and to correlate these microstructural parameters with clinical symptoms in patients with varying degrees of knee pain. A cross-sectional study was conducted on a cohort of 472 patients who presented with knee pain. DTI metrics, including mean diffusivity (MD), fractional anisotropy (FA), axial diffusivity, and radial diffusivity, were acquired from the articular cartilage of the knee. Participants were stratified into groups based on age, sex, and the Kellgren–Lawrence grade to assess the severity of osteoarthritis. The study found sex differences in DTI metrics, with males having higher MD (*P*-value = .12) and lower FA (*P*-value: .01) than females. Age-related increases in MD (*P*-value = .04) and decreases in FA (*P*-value: = .02) indicated collagen degradation in ECM with age. Kellgren–Lawrence grade positively correlated with MD (*P*-value < .001) and negatively with FA (*P*-value = .02), confirming DTI metrics’ link to cartilage pathology severity. No significant associations were found between DTI metrics and body mass index or smoking status. DTI was effective in evaluating the microstructure of collagen in cartilage ECM, indicating its metrics could be biomarkers for knee osteoarthritis severity. The results highlight DTI’s role in noninvasively assessing cartilage health and detecting early ECM degeneration.

## 1. Introduction

The intricate lattice of the articular cartilage extracellular matrix (ECM) is fundamental to joint function, conferring the capacity to withstand and distribute mechanical loads across the joints.^[1]^ Central to this function is the organization of collagenous fibrils, predominantly type II collagen, which forms a tightly packed network that is essential for maintaining the structural integrity and biomechanical properties of cartilage.^[2]^ Historically, the assessment of the microstructural organization of collagen fibrils within the ECM has been constrained to invasive methods, limiting the capacity for dynamic studies in living tissues or longitudinal monitoring of disease progression.^[3]^

Recent advancements in magnetic resonance imaging (MRI) techniques, particularly diffusion tensor imaging (DTI), have opened new avenues for the noninvasive study of soft tissue microstructure.^[4]^ DTI, a form of MRI that can characterize the diffusion of water molecules within tissue, has been extensively used in neuroimaging to map fiber tracts in the brain.^[5]^ Its application in articular cartilage is based on the premise that the diffusion patterns of water molecules are influenced by the ECM’s microarchitecture, including the orientation and density of collagen fibrils.^[6]^ By measuring the directional diffusivity of water, DTI metrics such as mean diffusivity (MD) and fractional anisotropy (FA) can provide insight into the integrity and organization of the collagen network.^[7]^

The potential of DTI in articular cartilage assessment has been underscored by a growing body of research linking alterations in DTI metrics to cartilage degeneration. Notably, changes in MD and FA have been associated with early degenerative changes in the ECM before gross morphological changes become evident on conventional MRI scans.^[8]^ Furthermore, the ability of DTI to assess cartilage at a microstructural level may offer a more sensitive means of detecting early osteoarthritic changes, monitoring disease progression, and evaluating the efficacy of therapeutic interventions.^[9]^

Despite these promising developments, the application of DTI in the characterization of articular cartilage ECM remains underexplored. The majority of studies have primarily focused on the brain or spinal cord, with only a handful delving into the potential of DTI in musculoskeletal imaging.^[10]^ Moreover, the relationship between DTI metrics and clinical characteristics such as knee pain severity, patient demographics, and lifestyle factors like smoking and body mass index (BMI) has not been thoroughly investigated.^[11]^

Therefore, the primary aim of this study was to characterize the microstructural organization of collagenous fibrils in the articular cartilage ECM using DTI and to explore the relationship between these microstructural parameters and clinical characteristics in a patient population.

## 2. Materials and methods

### 2.1. Study design

This observational study was designed to characterize the microstructural organization of collagenous fibrils in articular cartilage ECM using DTI. The study protocol was reviewed and approved by the Institutional Review Board of Fourth Hospital Wuhan and all participants provided informed consent.

### 2.2. Participants and sample size calculation

Participants were recruited from the outpatient clinic of the Department of Orthopaedics. Inclusion criteria were adults aged 18 to 65 with a clinical diagnosis of knee pain suspected to be due to early osteoarthritis. Exclusion criteria were a history of knee surgery, inflammatory arthritis, or contraindications to MRI. The sample size was calculated based on the primary outcome measure, which is the FA of collagenous fibrils in articular cartilage ECM. Assuming a standard deviation (SD) of FA values from preliminary data, a power of 80%, and an alpha level of 0.05 for detecting a clinically significant difference in FA, the sample size was determined using the following formula for a 2-sample comparison of means:


$$\begin{array}{l} n=2(\delta 2(Z\alpha /2\;\;\;+Z\beta )2\times (SD12\;\;\;+SD22)) \end{array}$$

where

- *n* is the sample size required per group
- 2*Zα*/2 is the *Z*-score corresponding to the desired alpha level (1.96 for 0.05 alpha level)
- *Zβ* is the *Z*-score corresponding to the desired power (0.84 for 80% power)
- 1*SD*1 and 2*SD*2 are the SDs of the 2 groups
- *δ* is the minimum difference of interest (effect size)

Assuming an effect size of delta, the calculated sample size per group was adjusted upwards to account for an estimated dropout rate of 10%. The calculated minimum sample size per group was determined to be 120, with adjustments for a 10% dropout rate bringing the total required sample size to 132 per group. Participants were recruited using a stratified sampling approach to ensure equal representation across key demographic variables such as sex and ethnicity. This design allowed for balanced subgroup sizes across these categories, as reflected in Table 1.

**Table 1:** Demographic and clinical characteristics of participation.

Variables	Total (n = 472)	Male (n = 236)	Female (n = 236)	*P*-value
Age (yr)				
Mean (SD)	45.2 (11.3)	44.8 (11.7)	45.6 (10.9)	.45
Range	18–65	18–65	18–65	N/A
BMI (kg/m^2^)				
Mean (SD)	27.5 (4.2)	27.8 (3.9)	27.2 (4.5)	.31
Range	18.5–39.9	19.0–39.5	18.5–39.9	N/A
Ethnicity				
Caucasian	300 (63.6%)	150 (63.6%)	150 (63.6%)	1.00
African American	72 (15.3%)	36 (15.3%)	36 (15.3%)	1.00
Hispanic	58 (12.3%)	29 (12.3%)	29 (12.3%)	1.00
Asian	30 (6.4%)	15 (6.4%)	15 (6.4%)	1.00
Other	12 (2.5%)	6 (2.5%)	6 (2.5%)	1.00
Duration of knee pain (yr)				
Mean (SD)	3.8 (2.1)	3.7 (2.0)	3.9 (2.2)	.56
Range	1–10	1–10	1–10	N/A
Kellgren–Lawrence grade				
Grade 0	94 (19.9%)	47 (19.9%)	47 (19.9%)	1.00
Grade 1	188 (39.8%)	94 (39.8%)	94 (39.8%)	1.00
Grade 2	150 (31.8%)	75 (31.8%)	75 (31.8%)	1.00
Grade 3	40 (8.5%)	20 (8.5%)	20 (8.5%)	1.00
Smoking status				
Nonsmoker	376 (79.7%)	188 (79.7%)	188 (79.7%)	1.00
Former smoker	58 (12.3%)	29 (12.3%)	29 (12.3%)	1.00
Current smoker	38 (8.1%)	19 (8.1%)	19 (8.1%)	1.00
Alcohol consumption				
Nondrinker	94 (19.9%)	47 (19.9%)	47 (19.9%)	1.00
Social drinker	302 (64.0%)	151 (64.0%)	151 (64.0%)	1.00
Regular drinker	76 (16.1%)	38 (16.1%)	38 (16.1%)	1.00

Data are presented as means ± SD or frequencies (%) unless otherwise noted.

BMI = body mass index, SD = standard deviation.

### 2.3. MRI acquisition

MRI scans were performed using a 3T MRI system (Model and Manufacturer) with a dedicated knee coil. DTI parameters were optimized for articular cartilage imaging, including a *b*-value of 800 s/mm^2^ and at least 6 diffusion-encoding directions.

### 2.4. DTI post-processing

Diffusion-weighted images were post-processed using (Specify Software) to generate DTI metrics, including FA, MD, radial diffusivity (RD), and axial diffusivity (AD). regions of interest were manually delineated on the articular cartilage in consensus by 2 musculoskeletal radiologists blinded to the clinical details. Diffusion-weighted images were post-processed using FSL (FMRIB Software Library, Version 6.0, Oxford, UK). Preprocessing included eddy current and motion correction using the eddy_correct tool. Diffusion tensors were fitted using the dtifit function to extract DTI metrics, including FA, MD, AD, and RD.^[12]^

### 2.5. Statistical analysis

Descriptive statistics were used to summarize demographic and clinical characteristics. DTI metrics were compared using independent *t*-tests. Multivariable linear regression analysis was used to adjust for potential confounders. Statistical significance was set at *P* < .05, and all analyses were performed using SPSS Version 22 (Chicago). Descriptive statistics were reported as means and SDs for continuous variables and as frequencies and percentages for categorical variables. Independent *t*-tests were applied to compare means between sexes, while ANOVA was used for comparisons across age groups. Multivariable linear regression analysis was conducted to evaluate the relationship between DTI metrics (dependent variables: MD, FA) and clinical predictors (age, BMI, Kellgren–Lawrence grade, smoking status). Post hoc pairwise comparisons were conducted using Tukey honest significant difference test to determine significant differences between groups. Chi-square tests was applied to compare proportions of categorical variables (e.g., smoking status, alcohol consumption) between groups. The enter method was used, with all predictors simultaneously entered into the model. Model performance was evaluated using the adjusted *R*^2^ and overall *P*-value of the regression model. Statistical significance was set at *P* < .05.

### 2.6. Ethical considerations

This study adhered to the Declaration of Helsinki guidelines. All participants provided written informed consent. Patient confidentiality was maintained by de-identifying all imaging data.

## 3. Results

### 3.1. Baseline characterization

Demographic and clinical characteristics are summarized in Table 1. Continuous variables, such as age, BMI, and duration of knee pain, are presented as means (SD), while categorical variables, such as smoking status and Kellgren–Lawrence grade, are presented as frequencies (percentages). The average age of participants was 45.2 years (SD = 11.3), with males and females showing no significant age difference (*P* = .45), both spanning ages 18 to 65. The mean BMI was 27.5 kg/m^2^ (SD = 4.2), with insignificant differences between genders (*P* = .31). Ethnic distribution was equal across genders, comprising 63.6% Caucasian, 15.3% African American, 12.3% Hispanic, 6.4% Asian, and 2.5% Other, showing no statistical difference (*P* = 1.00). The duration of knee pain averaged 3.8 years (SD = 2.1) without significant gender differences (*P* = .56). Kellgren–Lawrence grade distributions were identical between genders, indicating no significant difference in osteoarthritis severity. Smoking and alcohol consumption patterns showed no differences between genders (*P* = 1.00 for all groups) (Table 1).

### 3.2. Comparison of DTI metrics by sex

Table 2 indicates gender differences in DTI metrics. An independent *t*-test was used to compare MD between males (0.810 ± 0.050 × 10^−3^ mm^2^/s) and females (0.800 ± 0.045 × 10^−3^ mm^2^/s), The difference was not statistically significant (*P* = .129). FA, however, was significantly higher in females (0.300 ± 0.032) than males (0.290 ± 0.030), with a *P*-value of .014. AD and RD showed no significant differences between genders, with *P*-values of .285 and .048, respectively, although RD’s *P*-value suggested a marginally significant difference, warranting further investigation.

**Table 2:** Independent *t*-test comparison of DTI metrics by sex.

DTI metric	Male	Female	*t*-value	*P*-value
MD (×10^−3^ mm^2^/s)	0.810 (0.050)	0.800 (0.045)	1.52	.129
FA	0.290 (0.030)	0.300 (0.032)	−2.47	.014*
AD (×10^−3^ mm^2^/s)	1.220 (0.070)	1.210 (0.075)	1.07	.285
RD (×10^−3^ mm^2^/s)	0.620 (0.040)	0.630 (0.038)	−1.98	.048*

Results are reported as means ± SD with accompanying *P*-values from statistical tests.

AD = axial diffusivity, DTI = diffusion tensor imaging, FA = fractional anisotropy, MD = mean diffusivity, RD = radial diffusivity, SD = standard deviation.

* *P* < .05, indicating statistical significance.

### 3.3. Descriptive statistics for clinical characteristics by age of participation

Descriptive statistics across age groups revealed distinct patterns in clinical characteristics. The youngest group (18–35 years) reported an average BMI of 24.3 (SD = 3.1) and symptom duration of 3.5 years (SD = 1.8), with 12 participations (10%) smokers and 2 participations (1.7%) at Kellgren–Lawrence grade 3. The middle-aged group (36–50 years) had a higher average BMI of 27.6 (SD = 4.8) and longer symptom duration of 5.8 years (SD = 3.5), with 40 participations (20%) smokers and 25 participations (12.5%) at Kellgren–Lawrence grade 3. The oldest group (51–65 years) showed the highest BMI average of 28.9 (SD = 5.2) and longest symptom duration of 6.4 years (SD = 3.2), with 23 participations (15.1%) smokers and the largest share at Kellgren–Lawrence grade 3, 33 participations (21.7%) (Table 3).

**Table 3:** Descriptive statistics for clinical characteristics by age of participation.

Groups (yr)	n	BMI*	Symptom duration*	Smokers**	Kellgren–Lawrence grade 3**
18–35	120	24.3 (3.1)	3.5 (1.8)	12 (10%)	2 (1.7%)
36–50	200	27.6 (4.8)	5.8 (3.5)	40 (20%)	25 (12.5%)
51–65	152	28.9 (5.2)	6.4 (3.2)	23 (15.1%)	33 (21.7%)

BMI = body mass index, SD = standard deviation.

* Data reported as mean ± SD.

** Data reported as frequency and percentage.

### 3.4. Multivariable linear regression analysis for MD, FA according to adjusting for confounders

The multivariable linear regression analysis revealed that age and Kellgren–Lawrence grade were significant predictors of MD (adjusted *R*^2^ = 0.32, model *P*-value < .001). Specifically, age had a significant negative association with MD (B = −0.002, 95% CI = −0.004 to −0.0001, *P* = .045), while BMI and smoking status were not significant predictors. The Kellgren–Lawrence grade was a strong positive predictor of MD (B = 0.040, 95% CI = 0.028–0.052, *P* < .001) (Table 4). For FA, the adjusted *R*^2^ of the regression model was 0.25 (model *P*-value = .002), with age identified as a significant positive predictor (B = 0.0002, 95% CI = 0.00003–0.00037, *P* = .028). BMI and smoking status were not significant predictors for FA, while the Kellgren–Lawrence grade had a significant negative association with FA (B = −0.020, 95% CI = −0.033 to −0.007, *P* = .004) (Table 5).

**Table 4:** Multivariate regression analysis for MD.

Predictor	B coefficient	Standard error	ß value	*t*-value	*P*-value
Age	−0.002	0.001	−0.18	−2.01	.04*
BMI	0.0004	0.0003	0.12	1.33	.18
Smoking status (yes = 1)	0.015	0.01	0.15	1.50	.13
Kellgren–Lawrence grade	0.04	0.008	0.50	5.00	<.001*

BMI = body mass index, MD = mean diffusivity.

* *P* < .05, indicating statistical significance.

**Table 5:** Multivariate regression analysis for FA.

Predictor	B coefficient	Standard error	ß value	*t*-value	*P*-value
Age	0.0002	0.0001	0.20	2.20	.02*
BMI	−0.0001	0.0002	−0.05	−0.50	.61
Smoking status (yes = 1)	−0.008	0.009	−0.09	−0.89	.37
Kellgren–Lawrence grade	−0.02	0.007	−0.28	−2.86	.004*

BMI = body mass index, FA = fractional anisotropy.

* *P* < .05, indicating statistical significance.

### 3.5. Comparison of DTI metrics across different age groups

Table 6 illustrates the variance of DTI metrics across 3 distinct age groups. Significant differences in DTI metrics were observed among the 3 age groups (18–35, 36–50, and 51–65 years) using ANOVA (*P* < .001 for all metrics). Post hoc pairwise comparisons using Tukey honest significant difference revealed the following: MD and AD were significantly higher in the 51 to 65 group compared to both the 18 to 35 and 36 to 50 groups. FA was significantly lower in the 51 to 65 group compared to the 18 to 35 group. RD was significantly higher in the 51 to 65 group compared to both the 18 to 35 and 36 to 50 groups. These findings highlight age-related changes in DTI metrics, with the most pronounced differences observed between the youngest and oldest age groups.

**Table 6:** Comparison of DTI metrics across different age groups.

Age group (yr)	n	MD (×10^−3^ mm^2^/s)	FA	AD (×10^−3^ mm^2^/s)	RD (×10^−3^ mm^2^/s)
18–35	120	0.80 ± 0.046	0.31 ± 0.033	1.23 ± 0.065	0.61 ± 0.042
36–50	200	0.81 ± 0.052	0.295 ± 0.031	1.22 ± 0.072	0.62 ± 0.045
51–65	152	0.82 ± 0.049	0.280 ± 0.030	1.21 ± 0.078	0.63 ± 0.050
Total	472	0.81 ± 0.049	0.295 ± 0.031	1.22 ± 0.072	0.62 ± 0.046
*F*-value		8.56	6.21	7.34	9.02
*P*-value		<.001*	<.001*	<.001*	<.001*

Results are reported as means ± SD with accompanying *P*-values from statistical tests.

Values are reported as mean ± SD. Post hoc pairwise comparisons (Tukey HSD).

AD = axial diffusivity, DTI = diffusion tensor imaging, FA = fractional anisotropy, HSD = honest significant difference, MD = mean diffusivity, RD = radial diffusivity, SD = standard deviation.

* *P* < .05, indicating statistical significance between groups.

### 3.6. Correlation matrix of clinical characteristics and DTI metrics

Table 7 presents the correlation coefficients between clinical characteristics and DTI metrics. Statistically significant correlations (*P* < .05 or *P* < .01) are highlighted in the table. For example, age showed a moderate positive correlation with Kellgren–Lawrence grade (*R* = 0.40, *P* < .01) and a weak negative correlation with MD (*r* = −0.18, *P* < .05). Similarly, the Kellgren–Lawrence grade was strongly positively correlated with RD (*R* = 0.60, *P* < .01) and negatively correlated with FA (*r* = −0.28, *P* < .01). These findings underscore the relationships between clinical and imaging metrics, particularly the association of disease severity with changes in DTI metrics.

**Table 7:** Correlation matrix of clinical characteristics and DTI metrics.

Variable analyzed	Age	BMI	Symptom duration	Kellgren–Lawrence grade	MD	FA	AD	RD
Age	1							
BMI	0.12	1						
Symptom duration	0.25	0.37	1					
Kellgren–Lawrence grade	0.40	0.22	0.55	1				
MD	−0.18	0.04	−0.03	0.50	1			
FA	0.20	−0.01	0.02	−0.28	−0.45	1		
AD	−0.07	0.03	−0.01	0.40	0.75	−0.30	1	
RD	0.15	0.05	0.10	0.60	0.90	−0.60	0.65	1

Values represent Pearson correlation coefficients.

AD = axial diffusivity, BMI = body mass index, DTI = diffusion tensor imaging, FA = fractional anisotropy, MD = mean diffusivity, RD = radial diffusivity.

## 4. Discussion

The findings of this study have elucidated several significant associations and trends within the parameters of articular cartilage ECM as measured through diffusion tensor imaging (DTI) metrics, contributing substantially to the field of musculoskeletal imaging and characterization. The observed variance in DTI metrics across different age groups suggests an age-related evolution in the microstructural integrity of the cartilage. This age-related trend in the microstructural composition of cartilage has far-reaching implications for understanding the natural history of cartilage degeneration and potentially for establishing age-specific normative data for DTI metrics in healthy and diseased states.

The study’s revelation of sex-based differences in certain DTI metrics, particularly the higher FA in females, provides a novel understanding of the biological variances that may exist between males and females in the context of cartilage health. Although some metrics did not show significant differences, the borderline significance in RD between sexes indicates an area ripe for future investigation, possibly reflecting subtle sex-dependent variations in cartilage microstructure that could influence the development or progression of joint diseases. The complex interplay between clinical characteristics and DTI metrics, highlighted in the correlation matrix, signals a potential for DTI to serve as a biomarker for disease severity and progression. The negative association of age with MD and the positive relationship of age with FA were particularly noteworthy, suggesting that DTI could be a sensitive tool in capturing the nuanced changes in cartilage microstructure that occur with aging. The results further refined these insights, isolating age and Kellgren–Lawrence grade as significant predictors of both MD and FA. This underlines the potential of DTI metrics to serve as reliable indicators of cartilage health, with the ability to adjust for confounders and tease apart the contributions of various demographic and clinical factors. The comprehensive demographic and clinical characteristic data, with no significant differences in age, BMI, ethnic distribution, duration of knee pain, Kellgren–Lawrence grade, smoking status, and alcohol consumption patterns between genders, ensures that the study’s findings are robust and not confounded by these variables. The implications of these findings are manifold. Firstly, they suggest that DTI can serve as a noninvasive, quantitative imaging biomarker for the assessment of cartilage health, which could be used to monitor disease progression and response to therapy in clinical practice. Secondly, the study opens the door for the development of age and sex-specific diagnostic thresholds and intervention strategies. Thirdly, the data paves the way for future longitudinal studies to understand the dynamic changes in cartilage microstructure over time. Lastly, the study sets a precedent for the inclusion of DTI metrics in the comprehensive assessment of patients with joint pathologies, potentially aiding in the early detection of degenerative changes before irreversible damage occurs. DTI serves as a noninvasive tool to investigate the fibrous structure of tissues like muscle fibers. This imaging modality measures the directional diffusion of water and is useful for assessing the orientation and arrangement of muscle fibers.^[13,14]^ DTI provides quantitative data such as diffusion eigenvalues and FA, which relate to fiber tractography, including the length and orientation of fibers within muscles. Primary diffusion eigenvalues correspond to diffusion along the length of the muscle fiber, while secondary and tertiary eigenvalues are thought to be related to the cross-sectional size of the fibers.^[15–17]^ Despite its utility, the spatial resolution of DTI is limited and cannot capture details at the cellular level; hence, computational models are used to extrapolate microstructural information from DTI data.

Recent modeling efforts have been directed towards understanding diffusion within muscle tissues. A 2-compartment model that includes elliptically shaped muscle fibers within an extracellular matrix (ECM) was introduced, providing estimates of muscle fiber diameter that align with histological measurements from previous studies.^[11,17,18]^ The random permeable barrier model was developed to deduce microstructural features by fitting the time-dependent diffusion perpendicular to the fiber axis, recorded using a stimulated echo sequence with extended diffusion times.^[19]^ Application of the 2-compartment model to DTI data before and after unloading exercises suggested changes in muscle fiber characteristics, such as decreased permeability, reduced intracellular volume, and increased collagen, with a concurrent reduction in muscle fiber ellipticity due to the absence of mechanical stress.^[20]^ The random permeable barrier model has been used to analyze DTI data across different age groups, revealing age-related changes in muscle tissue, including alterations in the tertiary eigenvalue and a lower volume fraction in older muscles, which might indicate changes in membrane permeability with aging.^[21,22]^ While these models and their application to DTI data are still in exploratory stages, they offer valuable insights into the microstructural adaptations of muscle fibers and the surrounding ECM in response to aging and changes in mechanical loading. Sinha et al^[23]^ reviewed the application of advanced MRI techniques, including DTI, in studying the effects of aging and the remodeling of the extracellular matrix (ECM) on force transmission within the musculoskeletal system. While Sinha et al focused on a broader range of structural and functional MRI applications beyond DTI alone, their findings support our observation of age-related changes in the musculoskeletal system. Both studies suggest that aging significantly affects the ECM, which is consistent with the increase in MD and decrease in FA we observed in older individuals. Sinha et al emphasis on integrating MR findings with computational models to predict functional changes due to structural adaptations complements our results linking DTI metrics with clinical characteristics such as age and Kellgren–Lawrence grade. Yang et al^[24]^ utilized DTI to analyze the diffusion characteristics of water molecules in vocal fold scar tissue and correlated these with the textural characteristics of collagen fibers, using Pearson correlation analysis to establish quantitative relationships. Their findings of significant differences in FA and tensor trace values between scarred and normal tissue align with our study’s identification of differences in DTI metrics across age groups. Both studies highlight the sensitivity of DTI parameters to changes in tissue microstructure, though in different anatomical contexts. Ravindran et al^[25]^ discussed the development of chondrogenic ECM scaffolds for cartilage regeneration, with MRI used to track scaffold stability and chondrogenesis in vivo. The MRI characterization of these scaffolds showed mechanical properties and diffusion characteristics important for cartilage tissue regeneration. The Acknowledgments of MRI’s ability to noninvasively track changes aligns with our study’s use of DTI metrics to infer the integrity of knee joint structures. Both studies underscore the potential of MRI to assess and monitor musculoskeletal health and the progress of therapeutic interventions.

### 4.1. Strengths and limitations

The study provided insights into articular cartilage ECM’s microstructure but faced limitations. Its cross-sectional nature limited understanding of cartilage’s temporal changes, reducing the ability to infer causality. The selected cohort, excluding individuals with certain conditions or medications, might not fully represent the general population, affecting the findings’ generalizability. Despite adjustments for confounders, residual confounding could exist due to the study’s observational design. The Kellgren–Lawrence grade, used to assess disease severity, is prone to observer variability and might not detect early cartilage changes. DTI metrics, while innovative, are proxies for the complex cartilage structure, with their biological significance not fully understood. Motion sensitivity in DTI, despite motion correction efforts, could introduce artifacts. Age-related findings and sex differences noted require validation through longitudinal and larger multi-center studies to confirm trends and explore biological mechanisms. Volunteer selection bias might affect results, and factors like physical activity weren’t controlled for. Hormonal impacts, particularly in females, weren’t considered, which could influence cartilage health and DTI metrics. Clinical recommendations include establishing age-specific DTI metric ranges, considering age and symptom duration alongside Kellgren–Lawrence grade in osteoarthritis assessments, and recognizing the importance of early intervention. Sex-specific analyses are advised due to significant FA differences between genders. Observations on BMI, symptom duration, and Kellgren–Lawrence grade by age suggest weight management and lifestyle changes could benefit the elderly. Age and Kellgren–Lawrence grade were significant DTI metric predictors, overshadowing BMI and smoking status. However, the benefits of a healthy lifestyle remain undiminished. Gender did not significantly affect BMI, ethnic distribution, knee pain duration, or Kellgren–Lawrence grade distribution, suggesting these factors can be uniformly applied in clinical assessments for osteoarthritis across sexes.

## 5. Conclusion

Our study concluded that white matter integrity, as assessed by DTI metrics, declines with age, characterized by decreased FA and MD. Age and severity of knee osteoarthritis, indicated by the Kellgren–Lawrence grade, were significant predictors of changes in DTI metrics, suggesting that these factors are key in understanding and monitoring white matter health. While sex differences in FA were noted, other DTI metrics did not significantly differ between males and females, nor did BMI and smoking status show a significant impact on DTI metrics. The findings underscore the relevance of age and osteoarthritis severity in the assessment of neurodegenerative changes and support the potential of DTI as a biomarker in aging and osteoarthritis-related neurological research.

## Acknowledgments

The authors thank the rehabilitation staff for their support with this study.

## Author contributions

**Conceptualization:** Jie Liu, Xiaomu Tang.

**Supervision:** Jie Liu.

**Writing – original draft:** Jie Liu, Xiaomu Tang.

**Writing – review & editing:** Jie Liu, Xiaomu Tang.
